# THz Field Induced Second Harmonic Generation in Epsilon Near Zero Indium Tin Oxide Thin Films

**DOI:** 10.1021/acs.nanolett.5c02467

**Published:** 2025-08-01

**Authors:** Cormac McDonnell, Luca Carletti, Maria Antonietta Vincenti, Giuseppe Della Valle, Costantino De Angelis, Michele Celebrano, Tal Ellenbogen

**Affiliations:** † Department of Physical Electronics, Fleischman Faculty of Engineering, 26745 Tel-Aviv University, 69978 Tel-Aviv, Israel; ‡ Department of Information Engineering, University of Brescia, Via Branze 38, 25123 Brescia, Italy; § National Institute of Optics-National Research Council (INO-CNR), Via Branze 45, 25123 Brescia, Italy; ∥ Department of Physics, 18981 Politecnico di Milano, Piazza Leonardo da Vinci 32, 20133 Milano, Italy

**Keywords:** Indium tin oxide, Epsilon near zero, THz, Four wave mixing, Second harmonic generation, Nonlinear optics

## Abstract

Epsilon near zero (ENZ) thin films have attracted considerable attention due to their unique optical properties in the near-infrared (NIR), which have enabled a wide range of interesting phenomena and diverse applications. In nonlinear optics, the near-zero permittivity of films in the NIR region has been shown to enhance second-order nonlinear processes by several orders of magnitude, therefore boosting both second harmonic (SH) generation and broadband THz generation. In this work, we investigate THz-field-induced second harmonic (TFISH) generation in indium tin oxide (ITO) thin films in the ENZ spectral region. A symmetry-breaking electric field is applied using a high field strength broadband THz pulse, which when temporally overlapped with an ultrashort optical pulse, results in the emission of second harmonic from the centrosymmetric film. The experimental results mirror very well the predictions of a four-wave mixing optical model, capturing the interplay between linear and nonlinear effects driving the NIR-THz-ITO interaction.

## Graphical abstract

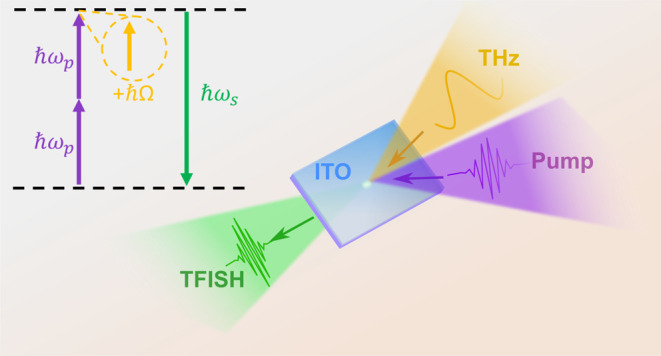

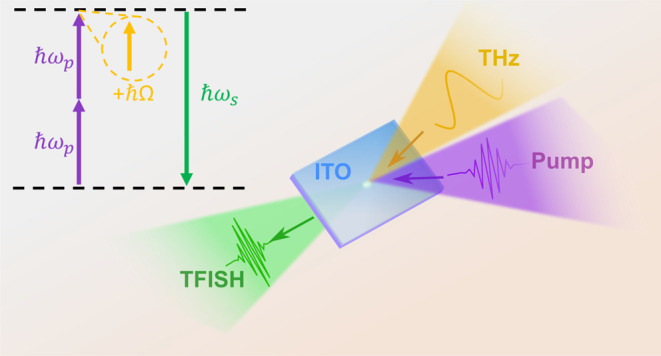

Indium Tin Oxide (ITO) thin films with high conductivity and high transparency in the visible spectral region^1^ have been exploited as a key material platform in industrial applications such as display devices,^2^ photovoltaics^3^ and light emitting diodes.^4^ Recent studies have shown that the optical properties of transparent conducting oxides (TCOs) in the near-infrared (NIR) spectral region, which are marked by a zero-crossing point of the real part of the dielectric permittivity, enable numerous applications in optics including pulse shaping,^5^ photonic time refraction^6^ and time cystals,^7,8^ ultrashort dynamics^9,10^ and enhanced nonlinear interactions.^11–14^ These phenomena occur around the near-zero permittivity spectral region, resulting in subwavelength confinement of the electric field in the thin film volume, enhancement of nonlinear effects and ultrafast modification of its dielectric properties.

Nonlinear field enhancement in this regime has been shown to increase the efficiency of various frequency conversion processes by orders of magnitude including second,^15^ third,^16^ high harmonic,^17^ and broadband THz generation.^18,19^ Second harmonic generation (SHG) from centrosymmetric materials such as ITO requires breaking of the local symmetry. This can be achieved by either using spatially structured radiation^20^ or light with a transverse magnetic (TM) incident polarization, leveraging surface effects,^21^ or designing specific meta-atom geometries to break the symmetry, such as split ring resonators or meta-atoms with C3 rotational symmetry.^22,23^ Symmetry breaking can also be induced by applying an external DC electric field, in a process known as electric field induced second harmonic generation (EFISH).^24–26^

EFISH is a third order nonlinear process,^24^ governed by the materials’ third-order susceptibility χ^(3)^. This is associated with four-wave-mixing phenomena, including third harmonic generation and the optical Kerr effect.^27^ EFISH was originally predicted theoretically in 1962 ^28^ and then demonstrated experimentally in calcite.^29^ Since then, EFISH has been observed in a variety of materials and configurations such as metal oxides,^30^ monolayers of WSe_2_,^31^ nanoantennas,^32^ ferroelectric single crystals^33^ and plasmonic nanocavities.^34^ Instead of using a static field to break the material symmetry, a low frequency, nonionizing THz pulse can provide the necessary symmetry breaking electric field in a process known as THz field induced second harmonic generation (TFISH), resulting in an all-optical control of the harmonic emission. This technique, first demonstrated in liquids^35^ and gaseous media,^36,37^ has been employed more recently in various centrosymmetric bulk materials with significant χ^(3)^ values such as silicon,^38^ quartz,^39^ plasma,^40^ diamond,^41^ and in 2D semimetals.^42^ Additionally, recent studies have shown that nanostructures can enhance the TFISH and third harmonic signals in silicon.^43^ This TFISH process can also be applied for broadband coherent THz detection, allowing detection of THz pulses with a high dynamic range using thin CMOS compatible devices.^44,45^

Here we present a theoretical and experimental investigation of TFISH in ITO thin films exhibiting an ENZ response in the NIR spectral region. We analyze the TFISH emission properties as a function of wavelength, polarization and intensity. A theoretical model for the dispersion of the effective χ^(3)^ for TFISH in the ITO film was developed and used in numerical simulations, allowing us to predict the experimental TFISH signal. Finally, we compare the TFISH signal to the SH from the same film at oblique incidence, revealing some key differences that allows to distinguish when this signal stems from an effective second order nonlinearity and the one associated with the TFISH process.

A general schematic of the TFISH process is shown in Figure 1a, consisting of the temporal and spatial overlap of a NIR femtosecond pulse with a broadband THz pulse in a 20 nm ITO thin film. In the experiment, the THz pulse was generated from a DSTMS organic crystal, which is phase-matched in the NIR region,^46^ resulting in the emission of a THz pulse with a peak field of 250 kV/cm (see Supporting Information S1 for a calculation of the conversion efficiency and S2 for the dependence of the THz field on the pump wavelength). Figure 1b shows the typical temporal and spectral characteristics of the emitted signal from the DSTMS crystal in the NIR region, consisting of a pulse with a fwhm duration of 100 fs with a spectral bandwidth up to approximately 6 THz. This agrees with the typical performance expected from this type of organic nonlinear crystal.^46,47^ The NIR pump central wavelength was tunable from 1250 to 1500 nm, delivering 50 fs pulses to the ITO sample with typical peak intensities used in the range of 5–15 GW/cm^2^. Typical second and third order nonlinear conversion efficiencies for ITO thin films in the GW/cm^2^ intensity range have been reported to be in the region of 10^–7^.^48^ Both the NIR pump and the THz pulse were normally incident onto the ITO thin film, i.e., the direction of propagation is normal to the ITO thin film surface plane. The resulting harmonic signal generated in the ITO thin film was transmitted through the glass substrate where it was collected and analyzed using a spectrometer. Appropriate spectral filters were used to isolate the spectral region of TFISH from the pump pulse, DSTMS harmonics and any harmonics from the laser cavity (see Supporting Information S3 for a full experimental description).

**Figure 1:**
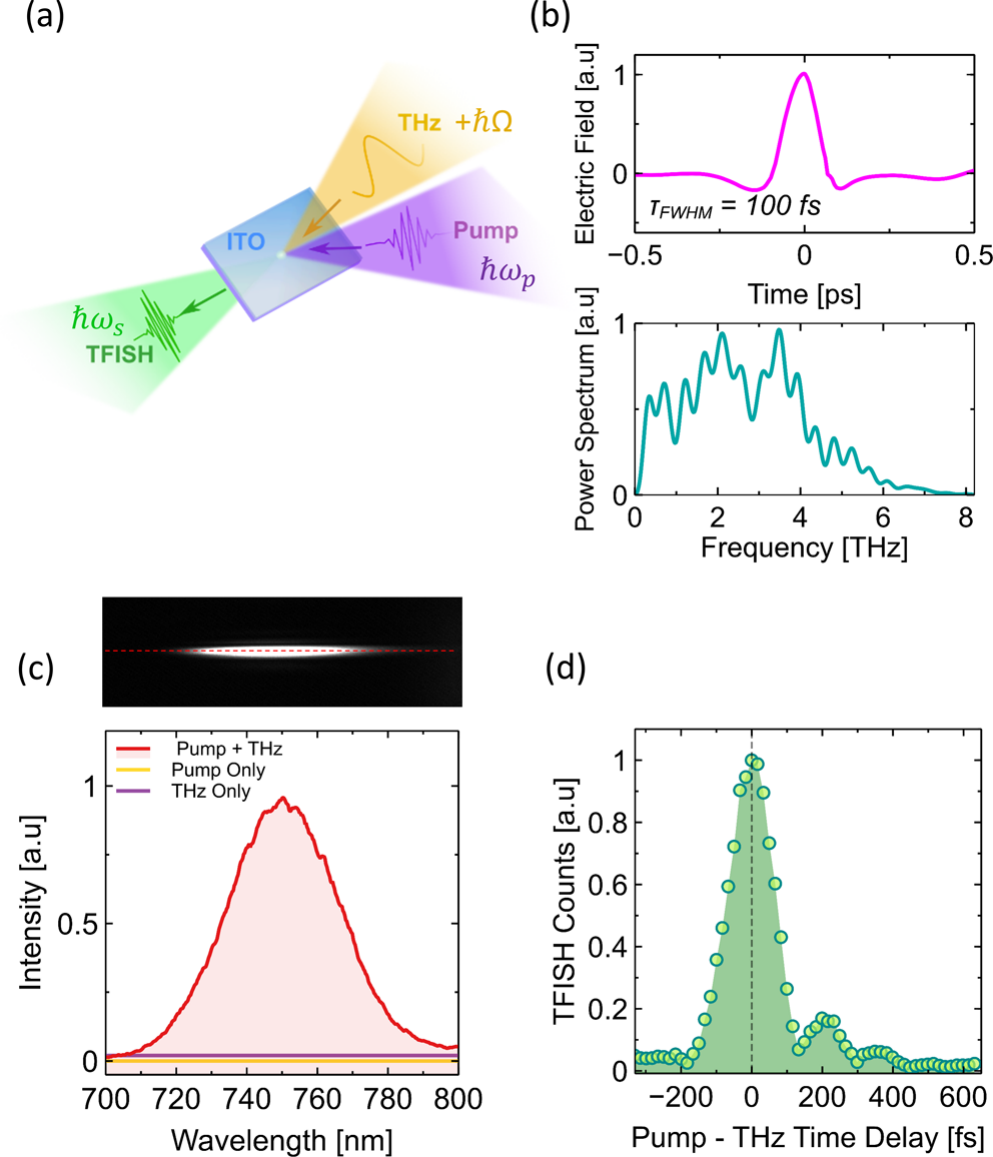
(a) Schematic of the experimental TFISH process: THz and optical pulses temporally and spatially overlap in an ITO film, generating a TFISH signal which is transmitted through the substrate. (b) Typical THz emission characteristics of the DSTMS organic crystal after pumping in the NIR, consisting of a near single cycle pulse with a fwhm pulse duration of 100 fs, with a corresponding frequency spectrum up to approximately 6 THz. (c) The TFISH signal is examined with respect to three different pumping conditions. The TFISH signal is only observed when the pump and THz pulse overlap on the ITO film. Top: Typical 2D distribution of the TFISH signal on the spectrometer CCD. The signal is lightly focused on the CCD to increase the SNR. (d) Harmonic signal as a function of the time delay between the pump and THz pulse.

The resulting signal from the ITO thin film was examined for three configurations at a pump wavelength of 1500 nm (the pumping wavelength for all experiments is set at 1500 nm unless stated otherwise); (i) only the pumping NIR pulse is incident on the sample (yellow line), (ii) only the THz pulse is incident on the sample (purple line), (iii) both the NIR and THz pulse temporally and spatially overlapped on the sample (red line), as shown in Figure 1c. As characteristic of TFISH, a harmonic signal emerges exclusively when the NIR and THz pulses overlap both temporally and spatially within the ITO layer. Furthermore, the harmonic signal of condition (iii) was further examined with respect to the inter pulse delay time between the pump and THz pulse, as shown in Figure 1d. The delay time was scanned in steps of 16 fs for a total time delay of 850 fs. The resulting harmonic signal reveals a clear time-dependent variation. A peak signal is observed, quickly falling off in a near symmetrical fashion with increasing and decreasing time delay. The zero-time delay is set at the position of peak TFISH emission. For positive time delays after the THz pulse, some asymmetry and further small oscillations can be observed. These features likely arise from reflections of the THz pulse throughout the optical setup and its interaction with air. The characteristics of the harmonic signal, requiring both temporal and spatial overlap of the pump and THz pulses, along with a distinct time-delay transient behavior, clearly confirm that the generated harmonic is a TFISH signal.

We developed a theoretical model to describe and predict the TFISH properties of the analyzed system. The nonlinear process in the ITO thin film is modeled as a degenerate four-wave mixing (dFWM) process. Figure 1a provides a schematic representation of the mixing process, where the NIR pump pulse has a frequency ω_*p*_ and the THz pulse has a spectrum of frequencies around Ω. The radiation generated by TFISH is defined as the signal with a frequency ω_*s*_ = 2ω_*p*_ ± Ω. The TFISH is characterized by the third-order nonlinear susceptibility tensor which has elements of the type χ_*ijkl*_^(3)^(ω_*s*_;ω_*p*_,ω_*p*_,Ω), where *ijkl* are Cartesian axes. The χ_*ijkl*_^(3)^ tensorial elements of the nonlinear susceptibility are derived assuming an isotropic response for ITO. The χ_*ijkl*_^(3)^ dispersion is derived applying the classical anharmonic oscillator model to an isotropic medium and summing the contributions from free electrons and bound electrons­(see Supporting Information S4);^49^ we thus write

1 $$\chi_{ijkl}^{\left(3\right)}\left(\omega_{s};\omega_{p},\omega_{p},\Omega \right)=K_{b}\left[\chi_{b}^{\left(1\right)}\left(\omega_{s}\right)\chi_{b}^{\left(1\right)}\left(\omega_{p}\right)\chi_{b}^{\left(1\right)}\left(\omega_{p}\right)\chi_{b}^{\left(1\right)}\left(\Omega \right)\right]\left(\delta_{ij}\delta_{kl}+\delta_{ik}\delta_{jl}+\delta_{il}\delta_{jk}\right)+K_{f}\left[\chi_{f}^{\left(1\right)}\left(\omega_{s}\right)\chi_{f}^{\left(1\right)}\left(\omega_{p}\right)\chi_{f}^{\left(1\right)}\left(\omega_{p}\right)\chi_{f}^{\left(1\right)}\left(\Omega \right)\right]\left(\delta_{ij}\delta_{kl}+\delta_{ik}\delta_{jl}+\delta_{il}\delta_{jk}\right)$$

where *K*_b_ and *K*_f_ are fitting parameters, χ_*f*_^(1)^(ω) and χ_*b*_^(1)^(ω) are the free-electron and bound electrons contribution to the linear susceptibility of ITO, respectively. The third order susceptibility χ_*ijkl*_^(3)^ tensor is thus derived from the linear susceptibility. The fitting parameters *K*_b_ and *K*_f_ incorporate both experimentally determined quantities and values taken from the literature (see Supporting Information S4).

Figure 2a shows the measured transmittance of the ITO thin film for TM- (orange circles) and TE- (blue squares) polarized incident light impinging at 45°. A pronounced decrease in the transmission is observed for TM-polarized waves around a wavelength of 1250 nm, indicating the ENZ spectral region. Both measured transmittance spectra are numerically reproduced using a transfer-matrix approach where the permittivity of the ITO thin film is characterized by a Drude-Lorentz model with one harmonic oscillator (see Supporting Information S5 for details of the permittivity model). The fitting parameters of the Drude-Lorentz model of ITO are chosen to reproduce numerically the measured transmittance spectra for TM and TE light as shown in Figure 2a. Figure 2b shows the ITO permittivity as a function of wavelength that was estimated by applying the described method. We can observe that the real part of the permittivity crosses zero at a wavelength of 1250 nm, as is typical for ITO. Next, applying eq 1 and using the fitted permittivity from Figure 2b, we calculate χ_*ijkl*_^(3)^(ω_*s*_;ω_*p*_,ω_*p*_,Ω) as a function of the TFISH emission wavelength at a fixed THz frequency of 2 THz, which is shown in Figure 2c. The third order susceptibility tensor is then used to analyze the wavelength dependent TFISH emission with fully vectorial numerical simulations implemented in COMSOL. Experimentally, the TFISH emission from the ITO film was measured for NIR pump wavelengths of 1300, 1400, and 1500 nm at a fixed pump intensity of 15 GW/cm^2^ and at a peak THz electric field of 250 kV/cm. The experimental results are shown as filled circles in Figure 2d. The measured spectra and relative intensity dependence of the TFISH in the NIR are reproduced numerically with fully vectorial simulations implemented in COMSOL where the fitting parameters *K*_b_ and *K*_f_ are tuned to reproduce the experiments. Figure 2c shows the estimated χ_*ijkl*_^(3)^(ω_*s*_;ω_*p*_,ω_*p*_,Ω) as a function of the TFISH signal wavelength with the fitting parameters *K*_b_ = 7.7121 × 10^–21^ s^6^A^2^/m^2^kg and *K*_f_ = 7.4423 × 10^–23^ s^6^A^2^/m^2^kg. We can observe that the real part of χ_*ijkl*_^(3)^(ω_*s*_;ω_*p*_,ω_*p*_,Ω) crosses zero at a wavelength of about 610 nm and monotonically decreases for higher wavelengths. This spectral behavior can be ascribed to the presence of the zero-crossing of the real part of the dielectric permittivity at the pump frequency (see Figure 2b).

**Figure 2:**
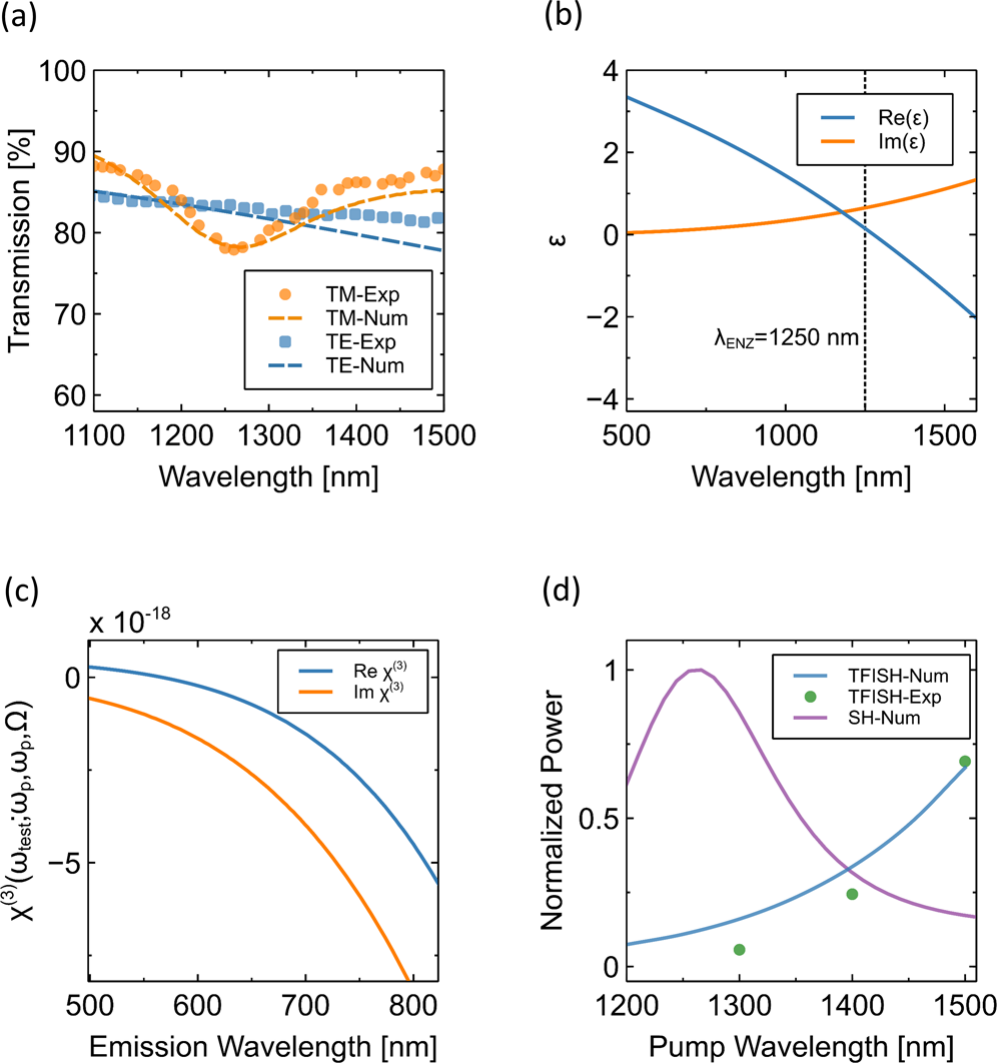
(a) Experimental and numerical transmittance of the 20 nm ITO thin film for TM and TE polarizations at an angle of incidence of 45°. A decrease in transmission is observed around 1250 nm for TM due to the ENZ crossing region. (b) ITO permittivity retrieved from the fitted transmittance. (c) Nonlinear susceptibility dispersion as a function of TFISH wavelength. The frequency of the THz radiation is 2 THz. (d) Experimental and numerical TFISH power as a function of wavelength, for a pump intensity of *I*_*p*_ = 15 GW/cm^2^. The TFISH emission was measured for NIR pump wavelengths of 1300, 1400, and 1500 nm at a fixed pump intensity of 15 GW/cm^2^ and at a peak THz electric field of 250 kV/cm. The results are overlaid with the numerically estimated SH power for the same pump intensity and for an angle of incidence of 50°.

Figure 2d compares the power of the numerically calculated TFISH signal with the numerically estimated power generated by the second-harmonic (SH) process in the ITO thin film. Since ITO is centrosymmetric, SHG can only be observed under oblique incidence and for a TM-polarized field.^50,51^ We compare the TFISH power with the maximum SH generated from the ITO thin film, which typically reaches peak emission for angles around 50° (see Supporting Information S6 for a description of the SH simulation and S7 for an experimental characterization of the SH emission). For a fair comparison between SH and TFISH, we fixed the NIR pump intensity to 15 GW/cm^2^, with the same film thickness of 20 nm for both configurations. As already shown in the literature for SHG in ENZ media,^15,52–54^ the peak emission is observed around the ENZ wavelength, with a following decrease in intensity with increasing wavelength. The theoretical model confirms that the TFISH signal measured for a NIR pump central wavelength of 1500 nm and normal incidence is comparable to the SHG signal obtained for a NIR pump central wavelength of 1250 nm and tilted 50° angle of incidence. Indeed, while the SHG process is enhanced by the ENZ condition, the TFISH is enhanced when spectrally detuned from this condition due to the dispersion of the corresponding third order nonlinear susceptibility (see Figure 2c).

The properties of the TFISH signal were further examined for several varying parameters such as the intensity of the incident radiation, NIR pump polarization, and angle of incidence. In the following analysis, both NIR pump and THz pulse beam are impinging at normal incidence onto the ITO thin film at a pump wavelength of 1500 nm. First, the effect of the NIR pump intensity on the TFISH signal was examined for a fixed THz field strength and is shown in Figure 3a. As expected, we observe a clear quadratic dependency between the TFISH signal and the NIR pump intensity. The effect of the THz field strength was then examined by increasing the THz electric field while keeping the NIR pump power constant, as shown in Figure 3b. The TFISH signal in this case increases approximately linearly with the THz field, in agreement with the nonlinear interaction described by the dFWM process.

**Figure 3:**
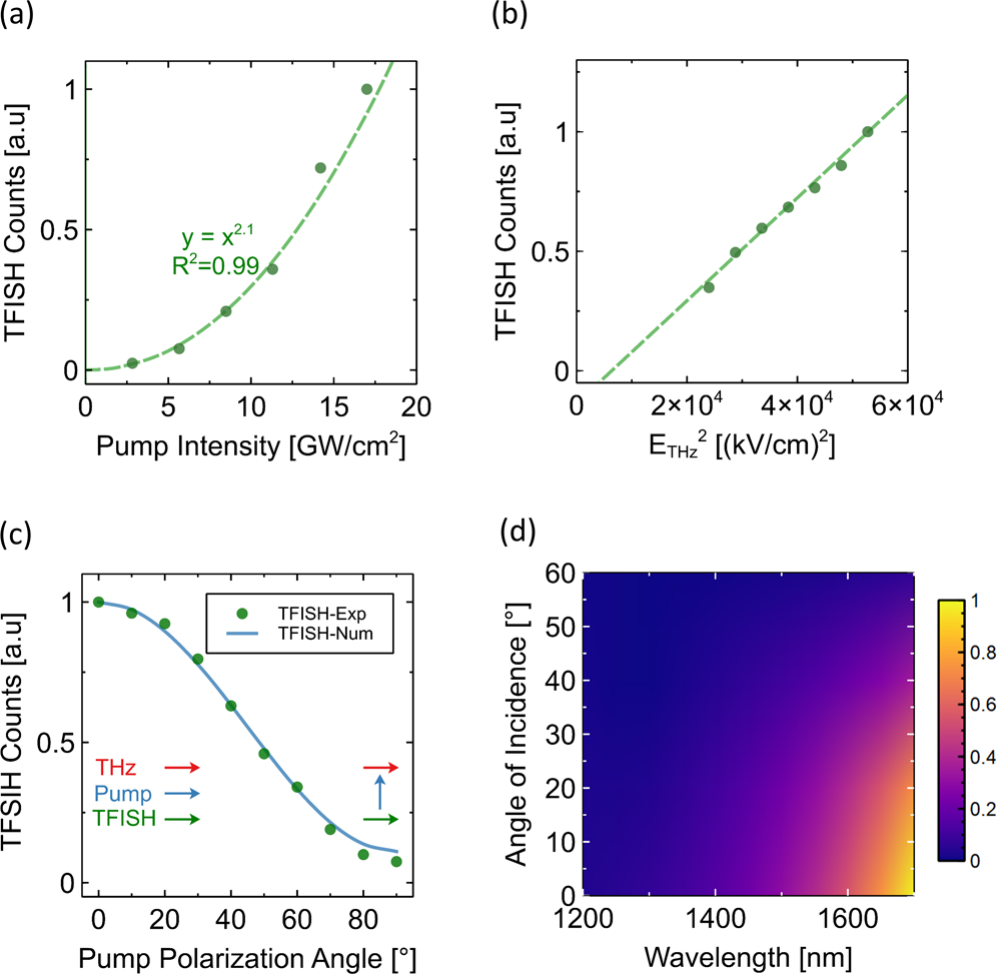
(a) TFISH signal as a function of NIR pump intensity with a fixed THz field strength, showing a second order dependence. (b) TFISH signal as a function of the THz electric field strength squared with a fixed NIR pump intensity *I*_*p*_ = 15 GW/cm^2^, showing a linear fit. (c) TFISH signal as a function of the angle between the pump and THz polarization; a maximum value is seen when they are copolarized. (d) Normalized TFISH power as a function of TM-polarized pump wavelength and angle of incidence.

The effect of the NIR pump polarization angle with respect to the THz polarization was then examined, as shown in Figure 3c. A maximum TFISH signal is observed when the NIR pulse and THz pulse are copolarized, with the resulting emission of a copolarized TFISH signal. As the pump polarization angle is rotated, the TFISH signal begins to decrease, reaching a minimum value when the polarizations of the NIR pump and THz beam are orthogonal to each other. The TFISH power estimated from our numerical model is superimposed over the experimental data in Figure 3c under the same conditions, confirming the excellent agreement between the measurements and the theoretical predictions. This polarization dependency follows from the isotropic nonlinear tensor χ_*ijkl*_^(3)^(ω_*s*_;ω_*p*_,ω_*p*_,Ω). Figure 3d shows the simulated TFISH power as a function of NIR TM-polarized pump wavelength and angle of incidence ranging from 0° up to 60°. We can observe that the TFISH signal is enhanced when the pump angle of incidence is 0° (i.e., normal incidence) and the central wavelength of the pulse is detuned from the ENZ condition. The former is a consequence of the type of nonlinear interaction which is based on the volume of the nonlinear material. The latter is in agreement with the observed dispersion of the χ_*ijkl*_^(3)^(ω_*s*_;ω_*p*_,ω_*p*_,Ω) shown in Figure 2c.

Finally, it is important to investigate ways to increase the TFISH efficiency, in order to make the TFISH process as relevant as possible for applications. In particular, it would be of interest to use lower applied THz fields, which do not require the use of high efficiency THz crystals and high peak power pulses. First, the TFISH efficiency can be significantly increased by using higher pump wavelengths, which in our study are limited to 1500 nm. This is due to the rapidly increasing χ_*ijkl*_^(3)^ for longer pump wavelengths (see Figure 2c). More interestingly, the TFISH emission may also be increased through the design and fabrication of plasmonic or dielectric nanostructure arrays on the ITO film, which through careful spatial engineering can result in further strong confinement of the optical and THz fields, resulting in an enhancement of the nonlinear interaction and conversion efficiency.

This study presents the first experimental demonstration of THz-field-induced second harmonic generation in an epsilon-near-zero ITO thin film, revealing distinct properties compared to conventional second harmonic generation. The experimental data shows very good agreement with a degenerate four wave mixing model, also confirmed by numerical simulations. Although TFISH achieves a nonlinear emission on magnitude similar to SH, its spectral dependence around the ENZ wavelength and angular dependence display unique characteristics that align with our theoretical predictions. Our findings establish a promising framework for exploiting ENZ materials in THz detection, TFISH emission and all-optical control of second harmonic generation. In contrast to commonly used bulk materials, subwavelength thin films as the one reported here are not bound to phase matching constrains and therefore can be exploited over a broad excitation and emission wavelength range.

## Supplementary Material



## Notes

Estimated DSTMS THz field strength, DSTMS pump wavelength dependence, experimental setup, model of the nonlinear susceptibility, ITO relative permittivity model, second harmonic generation in ITO thin films, and experimental ITO SH emission (PDF)
