# Supplementary material for: THz Field Induced Second Harmonic Generation in Epsilon Near Zero Indium Tin Oxide Thin Films
Source: Nano Lett. 2025 Aug 1;25(32):12201–6. doi: 10.1021/acs.nanolett.5c02467 (PMC12356113; doi:10.1021/acs.nanolett.5c02467)
Supplement: Supplementary file 1 [file nl5c02467_si_001.pdf]

Supporting Information: THz Field Induced Second Harmonic Generation in Epsilon Near Zero Indium Tin Oxide Thin Films

Cormac McDonnell[#*1], Luca Carletti[#2,3], Maria Antonietta Vincenti[#2], Giuseppe Della Valle[4], Costantino De Angelis[2,3], Michele Celebrano[4] and Tal Ellenbogen[1]

[1]Department of Physical Electronics, Fleischman Faculty of Engineering, Tel-Aviv University, 69978 Tel-Aviv, Israel

[2,3]Department of Information Engineering, University of Brescia, Via Branze 38, 25123 Brescia, Italy; National Institute of Optics–National Research Council (INO-CNR), Via Branze 45, 25123 Brescia, Italy

[4]Department of Physics, Politecnico di Milano, Piazza Leonardo da Vinci 32, 20133 Milano, Italy

S1: Estimated DSTMS THz Field Strength

In the TFISH experiments the maximum input power for the DSTMS pumping beam was approximately 250 mW. Using the beam parameters (f = 2 kHz, d_{pump} = 3 mm, τ = 50 fs) gives a peak intensity of ~71 GW/cm², an incident fluence of 3.5 mJ/cm² with a pulse energy of 125 µJ. The conversion efficiency of DSTMS is taken from the literature for a similar pump pulse intensity, (λ = 1000 nm, τ = 95 fs), giving a measured efficiency of approximately 1.8 %[1]. This leads to a converted THz pulse energy of 2.25 µJ for the input pulses used here. The peak THz electric field from the pulse energy is given as[1]

$$E_{THz} = \sqrt{\frac{W_{THz}}{2\epsilon_0 c\tau_{THz} A}} \quad \text{(S1)}$$

Where W_{THz} is the converted THz pulse energy, ϵ_0 is the permittivity of free space $(8.85 \times 10^{-12}$ $m^{-3}\ kg^{-1}\ s^4\ A^2)$, c is the speed of light, τ_{THz} is the FWHM pulse duration of the DSTMS pulse ($\tau_{THz}{\sim}100\ fs)$ and A is the beam area of the THz pulse. This will give a THz peak field of approximately 250 kV/cm.

S2: DSTMS pump wavelength dependence

The effect of the pumping wavelength on the generated THz field was examined for a constant incident pump intensity of 28 GW/cm². The emitted field strength variation is minimal in the NIR pump spectral region used.

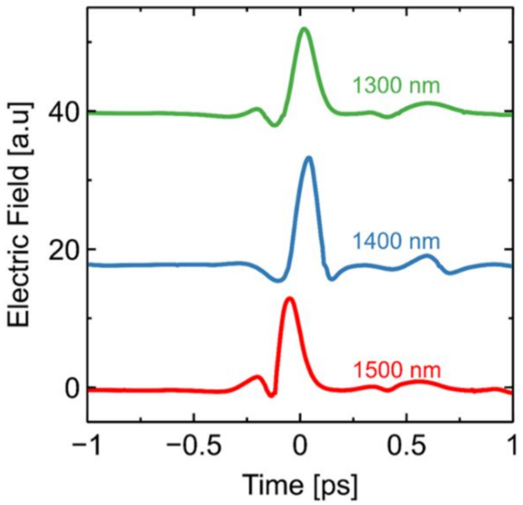

Figure S1 Time domain electric field traces of the emitted fields from the DSTMS crystal for pumping wavelengths of 1500, 1400 and 1300 nm respectively, showing a single cycle pulse for all pumping wavelengths.

S3: Experimental Setup

The laser source for the SH and EFISH experiments used an optical parametric amplifier (OPA) (Light Conversion TOPAS PRIME), with the relevant output wavelengths for the experiments from 1250 to 1500 nm. After emission from the laser the beam was split into two parts, a pump line for the ITO sample and a pump line for the nonlinear THz crystal. The THz crystal used was a DSTMS nonlinear organic crystal, which was pumped with a 3 mm output beam with a peak intensity of ~71 GW/cm². The generated THz pulse was collimated using an off axis parabolic mirror (f=50 mm). A second off axis parabolic mirror (f = 50 mm) was used to focus the THz pulse in a 4f system onto the ITO sample. On the second pump line the laser was directed through a hole in the second parabolic mirror where it was spatially and temporally overlapped with the THz pulse. Various optical elements on both pump lines were used to control the linear polarization angle and beam powers. The harmonics from the ITO sample were analyzed using an imaging spectrometer (Princeton Instruments Fergie). The THz beam path was placed in a box purged with dry air to reduce absorption of the generated THz pulse.

The temporal and spectral characteristics of the DSTMS organic crystal shown in Fig 1c were measured using a similar optical configuration. For this, the generated THz pulse was collected using the same 4-f system and then focused into a GaP nonlinear crystal with a thickness of 100 µm, where it was temporally and spatially overlapped with a probe pulse of 800 nm. Using a GaP crystal of this thickness allows detection of frequencies up to nearly 8 THz.

The linear transmission of the ITO thin film was measured using a super continuum white light source (NKT Photonics SuperK COMPACT) and a spectrometer (Ocean Optics NIR Quest).

S4: Model of the nonlinear susceptibility

The nonlinear response of ITO is modelled based on the classical harmonic oscillator (Lorentz model) where the nonlinearity is induced by a nonlinear restoring force that is acting on the electrons. We start from the equation of motion of the electron position, $\boldsymbol{x}$, driven by an electric field $\boldsymbol{E}$:

$$\ddot{\boldsymbol{x}} + \gamma\dot{\boldsymbol{x}} + \omega_0\boldsymbol{x} - b(\boldsymbol{x}\cdot\boldsymbol{x})\boldsymbol{x} = -e\boldsymbol{E}/m_0 \quad \text{(S2)}$$

where γ is the decay rate, ω_0 is resonant frequency, b is nonlinear coefficient, e is the electron charge, and m_0 is the electron mass. By applying perturbation theory, we obtain two separate equations that are associated with the linear and the nonlinear response respectively

$$\ddot{\boldsymbol{x}}_1 + \gamma\dot{\boldsymbol{x}}_1 + \omega_0\boldsymbol{x}_1 = -e\boldsymbol{E}/m_0 \quad \text{(S3)}$$

$$\ddot{\boldsymbol{x}}_3 + \gamma\dot{\boldsymbol{x}}_3 + \omega_0\boldsymbol{x}_3 - b(\boldsymbol{x}_1\cdot\boldsymbol{x}_1)\boldsymbol{x}_1 = 0 \quad \text{(S4)}$$

We seek steady-state solutions as

$$\boldsymbol{x}_3(t) = \boldsymbol{x}_3(\omega_s)e^{-i(\omega_s)t} \quad \text{(S5)}$$

Where the frequency of the TFISH signal is $\omega_s = 2\omega_p + \Omega$, with ω_p the frequency of the NIR pump and Ω the frequency of the THz radiation.

The nonlinear polarization at frequency ω_s is given by

$$\boldsymbol{P}(\omega_s) = -Ne\boldsymbol{x}_3(\omega_s) \quad \text{(S6)}$$

Where N is the electron density. Recalling the definition of third-order nonlinear susceptibility as

$$\boldsymbol{P}(\omega_s) = \varepsilon_0\boldsymbol{\chi}^{(3)}(\omega_s;\omega_p,\omega_p,\Omega)\vdots\boldsymbol{E}(\omega_p)\boldsymbol{E}(\omega_p)\boldsymbol{E}(\Omega) \quad \text{(S7)}$$

We obtain

$$\chi^{(3)}(\omega_s) = \frac{be^2}{3\omega_p^6 m_0}\chi^{(1)}(\omega_s)\left[\chi^{(1)}(\omega_p)\right]^2\chi^{(1)}(\Omega) \tag{S8}$$

Where $\chi^{(1)}$ is the linear susceptibility tensor calculated at ω_s, ω_p or Ω , e is the free-electron charge, m_0 is the electron mass and *b* is a pre-factor that depends on the specific material under investigation. We generalize Eq. S7 to both free and bound electrons and consider the total nonlinear susceptibility resulting from the sum of the individual contributions

$$\chi^{(3)} = \chi_b^{(3)} + \chi_f^{(3)}$$

$$\chi^{(3)} = \frac{b_b e^2}{3\omega_{pl}^6 m_{0b}}\chi_b^{(1)}(\omega_s)\left[\chi_b^{(1)}(\omega_p)\right]^2\chi_b^{(1)}(\Omega) + \frac{b_f e^2}{3\omega_p^6 m_{0f}}\chi_f^{(1)}(\omega_s)\left[\chi_f^{(1)}(\omega_p)\right]^2\chi_f^{(1)}(\Omega) \tag{S9}$$

Where the bound and free-electron contribution to the material susceptibility are given by

$$\chi_b^{(1)}(\omega) = -\frac{\omega_{pl}^2}{\omega^2-\omega_0^2+i\omega\gamma_l}; \qquad \chi_f^{(1)}(\omega) = -\frac{\omega_p^2}{\omega^2+i\omega\gamma_d} \tag{S10}$$

To fit the measurements, we used $b_L = \frac{\omega_0^2}{d^2}$ with d=0.15 nm, $b_D = b_L \times 5 \times 10^{-7}$, $m_{0b} = 0.01 \times m_0$, and $m_{0f} = 0.03 \times m_0$, where the scaling of the effective masses has been estimated in previous experiments [Ref. 48 in the main text].

S5: ITO Relative permittivity model

The relative permittivity of ITO used in Eq. (1) is obtained by fitting experimental data with a Drude contribution plus one Lorentzian oscillator defined as

$$\varepsilon(\omega) = 1 - \frac{\omega_p^2}{\omega^2+i\omega\gamma_d} - \frac{\omega_{pl}^2}{\omega^2-\omega_0^2+i\omega\gamma_l} \tag{S11}$$

Where ω_p is the plasma frequency, γ_d is decay rate, and ω_{pl},ω_0, γ_l are plasma frequency, resonant frequency and decay rate of the Lorentzian oscillator, respectively. The values of all the terms is reported in Table S1.

Table S1. Parameters used to fit the ITO permittivity to the experimental data from Figure 2a.

ω_p (rad/s)	γ_d (rad/s)	ω_{pl} (rad/s)	ω_0 (rad/s)	γ_l (rad/s)
2.9000×10^{15}	1.6000×10^{14}	2.1662×10^{16}	1.3186×10^{16}	1.8837×10^{13}

S6: Second harmonic generation in ITO thin films

As ITO is a centrosymmetric material, second-harmonic generation arises primarily from the free-electron components, which include surface, magnetic (via the Lorentz force), and convective contributions, as detailed in Ref. 50. Figure S2 shows the transmitted SH from a 20nm ITO film fitted with the parameters in Table S1. SH shows the characteristic teardrop shape associated with the nonlinear processes occurring in ENZ materials with a maximum of conversion efficiency occurring in proximity of the ENZ region for angles of incidence that increase with decreasing thicknesses. For these calculations the effective mass of the free-electrons in ITO is assumed to be $m_e^* = 0.033 m_e$, as previously reported in Ref. [48] in the main text, while the plasma frequency and damping coefficient are identical to those used for the linear fit (see Table S1).

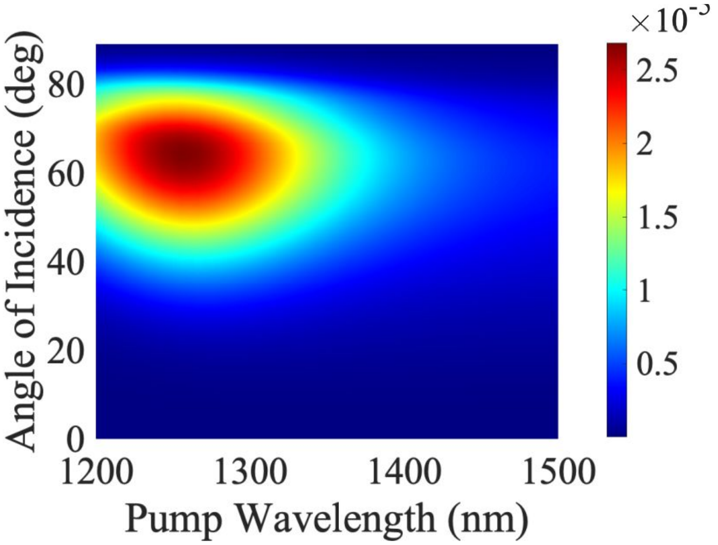

Figure S2. Numerical calculation of the transmitted SH signal from a 20nm ITO film with dielectric permittivity described with the parameters in Table S1 as a function of pump wavelength and angle of incidence. The intensity of the pump is I_p= 15 GW/cm².

S7: Experimental ITO SH emission

Some of the SH emission properties of the ITO thin film are shown below with in Fig S3. Fig S3a a shows the SH intensity as a function of the pump angle of incidence. As expected, there is no emission observed for pulses incident at 0°, with increasing the angle of incidence resulting in appreciable SH emission. Fig S3b shows the peak SH emission as a function of the incident pump pulse intensity and as expected the trend shows a second order dependence.

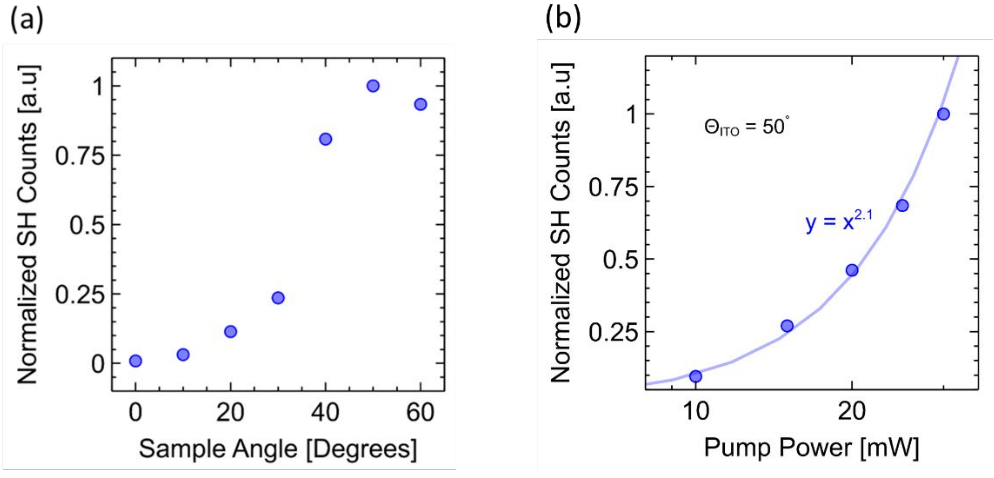

Figure S3 (a) SH signal as a function of sample angle for a TM polarization configuration (b) SH counts as a function of pump power, showing a second order dependence

References

(1) Zheng, Z.; Wang, K.; Li, H.; Meng, X.; Tian, Y.; Song, L. High-Repetition-Rate Strong-Field Terahertz Source by Optical Rectification in DSTMS Crystals. *High Pow Laser Sci Eng* **2024**, *12*, e61. https://doi.org/10.1017/hpl.2024.50.
